# Effects on Heart Rate Variability of Stress Level Responses to the Properties of Indoor Environmental Colors: A Preliminary Study

**DOI:** 10.3390/ijerph18179136

**Published:** 2021-08-30

**Authors:** Jiyoung Oh, Haengwoo Lee, Heykyung Park

**Affiliations:** 1 Institute of Ecology, Pusan National University, Busan 46241, Korea; ojy1245@pusan.ac.kr; 2 College of Design, Sangmyung University, Cheonan-si 31066, Korea; 2hw@smu.ac.kr; 3 Department of Interior Architecture, Inje University, Gimhae-si 50834, Korea

**Keywords:** environmental color, heart rate variability, stress group, indoor space color

## Abstract

Color is the most potent stimulating factor affecting human vision, and the environmental color of an indoor space is a spatial component that affects the environmental stress level. As one of the methods of assessing the physiological response of the autonomic nervous system that influences stress, heart rate variability (HRV) has been utilized as a tool for measuring the user’s stress response in color environments. This study aims to identify the effects of the changes of hue, brightness, and saturation in environmental colors on the HRV of two groups with different stress levels—the stress potential group (*n* = 15) and the healthy group (*n* = 12)—based on their stress level indicated by the Psychosocial Well-being Index (PWI). The ln(LF), ln(HF), and RMSSD values collected during the subjects’ exposure to 12 environments colors of red and yellow with adjusted saturation and brightness, were statistically analyzed using t-test and two-way ANOVA. The results show that the HRV values in the two groups did not significantly vary in response to the changes in hue, brightness and saturation. The two groups’ stress factors distinguished according to the stress levels by the PWI scale affected the In(LF) parameter, which demonstrates that the PWI index can be utilized as a reliable scale for measuring stress levels. The ultra-short HRV measurement record and the use of a sole In(LF) parameter for stress assessment are regarded as the limitations of this study.

## 1. Introduction

Color is an essential visual component that influences our lives and creates physiological and psychological differences in spatial perception [1,2]. Even when seeing the same color, various emotions are felt depending on the individual’s color preference, cultural factors, and so forth, and colors are also perceived as positive or negative according to the individual’s stress and psychological state [3,4].

Nowadays, numerous individuals suffer from severe stress disorders that directly influence their physiological and psychological status [5]. A study reported that the number of patients with mental disorders increased by 67% from 2015 to 2019, and 70% of internal medicine department visits were due to stress [6]. Additionally, the incidence rate of depression due to college students’ stress was approximately 24% [7]. Depression can negatively affect interpersonal relationships, daily activities, and academic performance, and worsening depression can increase the frequency of high-risk behaviors such as suicide attempts. The COVID-19 pandemic prevention measures have required social isolation, leading to a marked increase in time spent indoors; thus, the development rate of related mental illnesses, including stress, anxiety, and depression, has increased [8,9]. 

Of the elements comprising the spatial environment, environmental color exerts the most potent influence, stimulating human vision. Thus, studies investigating how environmental color affects individuals’ stress levels are necessary. Studies have regularly mentioned the lack of evidence-based research in this area [10,11] and encouraged additional empirical research to elucidate the effects of environmental colors on individuals’ stress levels. 

Heart rate variability (HRV) represents the variation in heart rate signal and can be calculated by the time interval between two adjacent R waves. The variation reflects time-varying influences of the autonomic nervous system (ANS) [12]. Measuring HRV is a relatively reliable method for evaluating stress-induced physiological responses [5] and a noninvasive biomarker measure that can quantify stress level because it reflects ANS activity [13]. 

Therefore, this study aimed to investigate the effects of the environmental colors’ hue, brightness, and saturation on the HRV in study groups defined by stress level. The level of environmental stress in subjects was investigated and analyzed to identify particular environmental color conditions that may negatively impact individuals’ stress levels. In this study, 27 subjects in their 20s who were identified to be vulnerable to stress but had healthy visual conditions and physical activities were included, and their HRVs were measured in color environment. 

Our research findings could be used as empirical evidence for establishing pleasant color environments for indoor spaces.

### 1.1. Environmental Color and Stress Response

According to Jonauskaite et al. [3], dwellers of indoor spaces are affected by the color of the indoor space while resting; the authors clinically verified that the environmental color might effectively reduce actual stress and anxiety levels. Thus, dwellers’ exposure to environmental color throughout their daily activities affects their stress, predictably leading to stress responses. The physiological responses to stress include blood pressure increase, heart rate increase, respiratory rate increase, difficulty breathing, sensory abnormalities, muscle tension, increased pain sensitivity, gastrointestinal symptoms, and allergic reactions [5].

In this study, we reviewed the literature on the effects of environmental color or lighting on individuals’ stress-induced physiological responses and then used the findings as evidence for designing the experiment. Gerard [14] and Ali [15] conducted experiments to verify the effects of color light stimulation on electroencephalogram (EEG) and found that the recovery of alpha waves in EEG was more significant for red and blue colors. Nakshian [16] and Wilson [17] found a greater degree of electrodermal response hand tremors for red than green, indicating that the red color environment stimulates the ANS more significantly. Nourse and Welch [18] measured the skin conductivity response—subject to a change in ANS activity—to 6-minute-long color light stimulation by exposing 14 participants to 1 min of green light and 1 min of purple light and found that the skin conductivity was higher for purple light than green light. Jacob and Hustmyer [19] found that a 1 min exposure to red, yellow, green, and blue lights on a screen affected skin conductivity but did not affect heart rate or respiration significantly. The most significant arousal was observed for red, followed by green, yellow, and blue. Hamid and Newport [20] verified that children’s hand strength was affected by the color of the room. The hand strength increased in a pink room, decreased in a blue room, and decreased even more in a gray room. Abbas et al. [21] demonstrated that subjects’ heart rates changed 2 min after being exposed to the light of a different type of color. They found that the heart rate increased during the exposure to red but slightly decreased during the exposure to blue. In a study on how six colors with different hues and brightness levels affected the reading, emotional evaluation, and heart rate of the users of a personal study space, Al-Ayash et al. [22] verified that reading efficiency was promoted more by vivid colors than pale colors. Additionally, red and yellow increased heart rate, and blue decreased it. 

These research results regarding the physiological response to environmental color stimulation suggest that warm colors such as red and yellow stimulate the ANS to increase heart rate and skin conductivity response. By contrast, cool colors such as green and blue relax the ANS to reduce them. 

This study describes how individuals’ stress levels are affected by colors—primarily warm colors, such as red and yellow. These warm colors have been studied as stress stimuli in various studies, including those of Gerard [14], Ali [15], Nakshian [16], Wilson [17], Jacobs and Hustmyer [19], Hamid and Newport [20], Abbas et al. [21], and Al-Ayash et al. [22]. However, experiments have been conducted to evaluate color lighting of primary colors that are not readily applicable to realistic environments, making it challenging to apply such research results to realistic settings. HRV analysis in subjects under different color environment in the context of color stimuli in the range of colors applied to a realistic environment would help researchers derive more empirical research results. 

### 1.2. Stress and Heart Rate Variability

**Figure 1:**
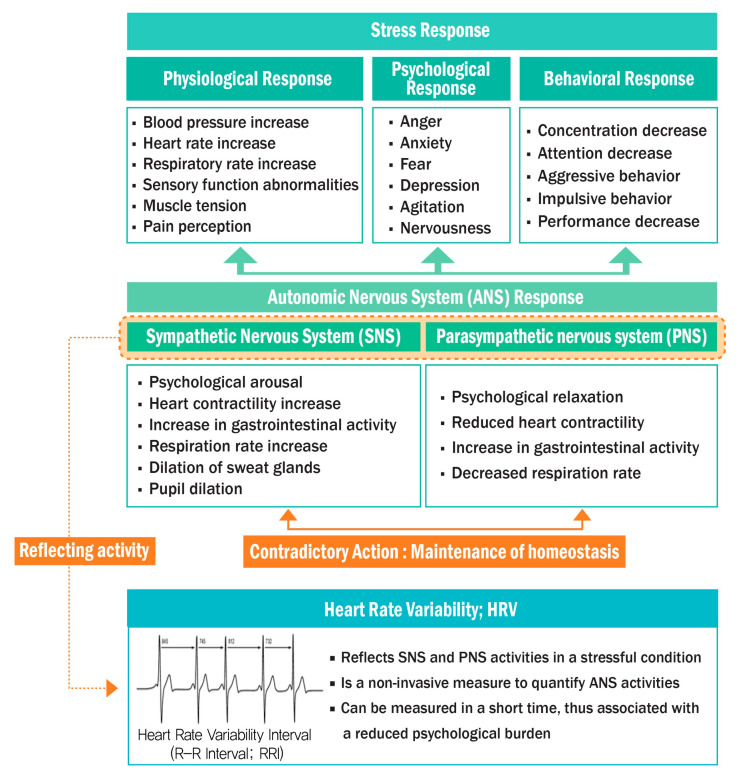
Relationships between stress, ANS, and HRV.

Stress induces physiological, psychological, and behavioral responses caused by ANS stimulation. Stress responses are observed amid the contrasting actions of the sympathetic nervous system (SNS) and parasympathetic nervous system (PNS), which form the ANS. HRV reflects the related activity level [23] and is an index of the time-varying differences between two adjacent heartbeats that have been used for the objective evaluation of mental health and stress [24]. Figure 1 describes stress-induced physiological, psychological, and behavioral reactions manifested by SNS and PNS interactions with ANS stimulation. The relationship between HRV and the ANS is illustrated in Figure 1. 

Analytical methods of HRV vary, and the most common methods are time-domain analysis and frequency-domain analysis. The time-domain analysis computes the time intervals between two adjacent heartbeats within normal limits. It is advantageous for long-term monitoring but limited because it does not quantify ANS balance and cannot differentiate SNS activity from PNS activity [25]. An HRV signal can be considered as the superposition of different frequency components. The frequency-domain analysis decomposes the HRV signal into the components of various frequency bands for further analysis. Frequency-domain analysis can separate the SNS and PNS activities, facilitating the physiological interpretation of HRV. This method is preferred for short-term measurements of less than 5 min [26]. The Task Force of the European Society of Cardiology and the North American Society of Pacing and Electrophysiology provides HRV measurement and analysis guidelines. The definitions of the parameters derived from the time-domain and frequency-domain analysis methods are listed in Table 1 [26]. 

HRV measurement is noninvasive and can be performed relatively quickly, providing a significant benefit: the measurement is convenient and efficient. However, contradictory or inconsistent results have been reported in the relationship between the HRV parameters and stress [5,27]. Therefore, we cannot conclude that specific parameters of HRV represent stress, but we can infer the stress states by considering and referencing several types of parameters. Kim et al. [24] identified the HRV parameters highly correlated with stress in their literature review of the HRV parameters and stress. They found that the characteristic relationship decreased in high-frequency (HF) components and increased in low-frequency (LF) components. Despite the fact that changes in parameters such as a decrease in HF and increase in LF are not considered representatives of the stress states, we plan to analyze parameters of HF and LF in this study based on the suggestion of Kim et al. [24]. Additionally, we plan to compare states by analyzing RMSSD parameters, which are known to reflect HRV status in short-term measurements of <5 min. [12,26]. In the studies listed in Table 2, color lighting was used as the experimental variable, brightness was observed in varying degrees, and a relatively narrow range of colors such as red, green, and blue was used. The color lighting conditions that were used cannot be easily applied to realistic settings because these studies were case studies conducted in the context of color therapy. They verified a change in HRV due to color stimulation, but the color conditions are far from applicable to a realistic environment. Thus, the ability to apply the research results described in Table 2 to actual environments may be limited. This study used environmental colors applicable to the actual environment for experimental stimulation; thus, we expect to derive empirical study results. This study differs from those in the literature: we set up an experiment space similar to a realistic color environment to verify the influence of daily color environments on individuals’ stress levels, whereas researchers have used color lighting in the context of color therapy as the study variable. 

## 2. Methods

**Figure 2:**
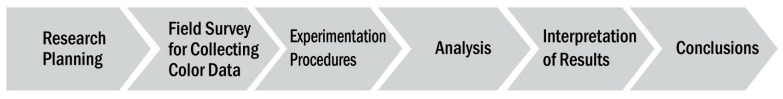
Study design.

As shown in below Figure 2, the study was designed as follows: study plan establishment, on-the-spot survey to health facilities for color data collection, HRV measurement experiment, data analysis of HRV, result analysis, and conclusion. The details of each study designs are delineated in the following chapter.

### 2.1. Configuration of Colors

According to the Organization for Economic Co-operation and Development’s (OECD’s) Health Statistics for 2020 [33], of all OECD countries, South Korea has the most public health environmental facilities, such as patient beds and medical devices, and the most outpatient visits per capita. Thus, the mortality rates of significant diseases in South Korea are relatively lower than those of other OECD countries. Additionally, in Asia, South Korea is a significant medical tourism host because of the high-quality medical services and medical facilities and the government-driven effort toward customer-centered quality improvement [34]. Nonetheless, the stress level in South Korea is higher than that in Japan, China, the United States, England, and Germany [35]; health care professionals’ stress levels are high [27,36] and have been worsening during the COVID-19 pandemic [37]. 

In this study, 544 sets of environmental color data were measured with a spectrophotometer (Minolta CM-2500d, Konica Minolta, Osaka, Japan; Table 3). The data were obtained from 22 public health facilities (8 large general hospitals with >1000 patient beds, 8 nursing hospitals, and 6 public health clinics (Table 4)) in which health care professionals had exceptionally elevated stress levels. 

We created the color stimuli for our experiment’s environment to be within the range of realistic environmental colors based on our collected color data. They were divided into dominant, complementary, and accent colors according to the intensity applied to each facility. The results from the analyses are listed in Table 5. 

**Figure 3:**
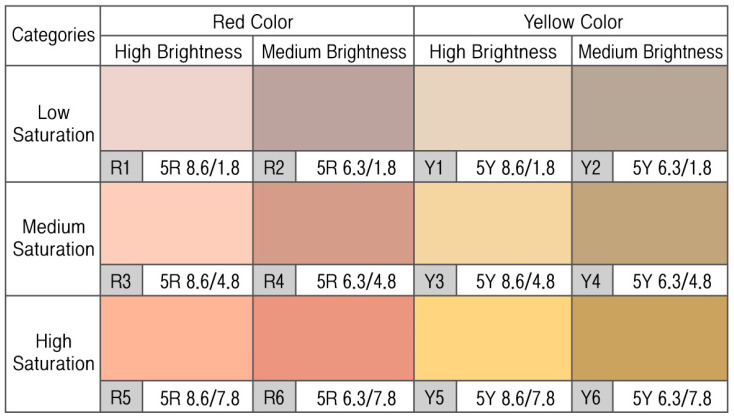
Color samples used for the experiment on the HRV of different stress groups.

With the color of a wall that was the most sizable visual area as the reference, the average brightness and saturation values were used as the reference values for the test stimulus; the red and yellow hues were selected based on our review described in Section 1.1. High brightness at 8.6 and medium brightness at 6.3 were selected as the reference, and these numbers were the average brightness values of a dominant color and accent color, respectively. The reference for saturation was chosen by starting from the average saturation of the dominant color at 1.8 and increasing it by 3 degrees; the low, medium, and high saturation levels were 1.8, 4.8, and 7.8, respectively. The color samples applied to our experiment regarding the HRV for different stress groups are displayed in Figure 3. 

### 2.2. Participants

The subjects who participated in the experiment are a total of 27 people comprised of 14 men and 13 women, whose average age is 22. Participants in our study volunteered to participate after being informed of the experiment by online or offline notifications. Participants were excluded if they were smokers, claustrophobic, mentally ill, obese, or suffered specific chronic illnesses that could affect their ANS. They were asked to abstain from consuming alcohol and take a >6 h nap before the experiment. Additionally, caffeine consumption was restricted 3 h before the test [38], and the Ishihara test was administered to the participants for testing color blindness or color weakness [3]. Before the experiment, the subjects were directed to conduct a self-diagnosis of their health, and they were found to have a good health condition without any particular health problems. According to a mental illness survey [39], mood disorders, depressive disorders, and anxiety disorders are most prevalent in individuals aged between 20 and 29 years, and this generation could be most vulnerable to stress. This phenomenon is not limited to South Korea. The American Psychological Association reported that individuals in the United States aged between 20 and 29 years have the highest stress levels [40]. Therefore, the 20s are considered an age range in which individuals are vulnerable to stress, and because this is a preliminary study, we focused on testing people in their 20s first. Additionally, this group generally has satisfactory vision for perceiving colors; thus, we deemed them the generation with the best physical vitality who could demonstrate the most precise physiological responses, including HRV, to a slight change in environmental colors. 

To figure out the stress levels of the subjects and sort the experimental group, the stress level of subjects was assessed with the Psychosocial Well-being Index (PWI) scale [41]. The PWI we used was a modified Korean version, comprising the 45 items selected from the 60 items of Goldberg’s General Health Questionnaire that had the highest reliability [42]. Its reliability and validity have been confirmed, and it is commonly used to assess levels of daily stress [43,44]. This scale comprises questions on four factors: (1) depression and anxiety, (2) societal role performance and self-trust, (3) sleep disorders and depression, and (4) general well-being and vitality. The PWI is a survey that uses a 4-point Likert scale, and the total score was used to divide the sample into the high-stress group (>63), potential stress group (23–62), and healthy group (<23). On the basis of the PWI scores of this study’s participants, 15 belonged to the stress group (male 6, female 9) with an average score of 40, and 12 belonged to the healthy group (male 8, female 4) with an average score of 15. Considering the significant difference between the two groups, which was greater than two-fold, the stress levels were also somewhat different.

### 2.3. Experimental Environment

**Figure 4:**
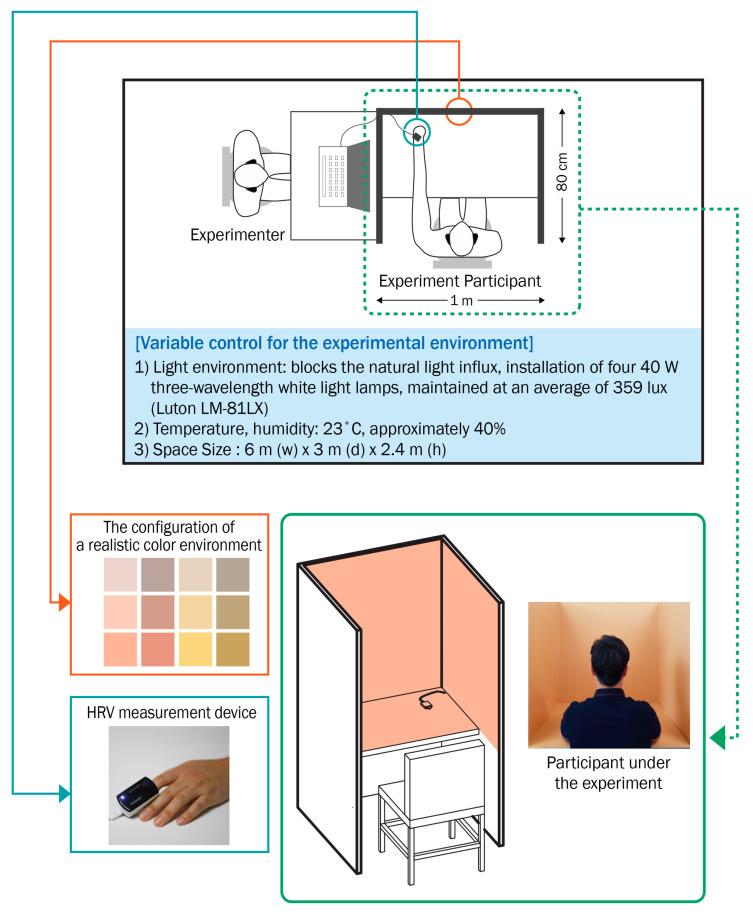
Experimental environment.

In Figure 4, the experimental environment was configured with artificial lighting (Table 6, Osram FHF32SS EX-D, 32W, four ramps, Osram, Munich, Germany) after shutting off the natural light influx. The illuminance was measured by an illuminance meter (Table 7, Luton LM-81LX) twice on the day of the experiment. The average illuminance measurement was 359 lux [22]. The experimental space floor, walls, and ceiling were finished with achromatic white matte paint. Its dimensions were 6.0 m (w) × 3.0 m (d) × 2.4 m (h). The color space of the HRV measurement was just big enough to block one seated participant’s vision completely where the color stimulus material of 1.0 m (w) × 0.8 m (d) × 1.2 m (h) was placed. The experimental environment was designed to be as similar to the space of daily activities as possible; thus, it did not include any additional local light source. The illuminance of the space was 250–500 lux, a commonly found level for office space [45]. It was designed to receive the ceiling light, and there was no other color stimulus from the ceiling [34,46].

### 2.4. Procedures

**Figure 5:**
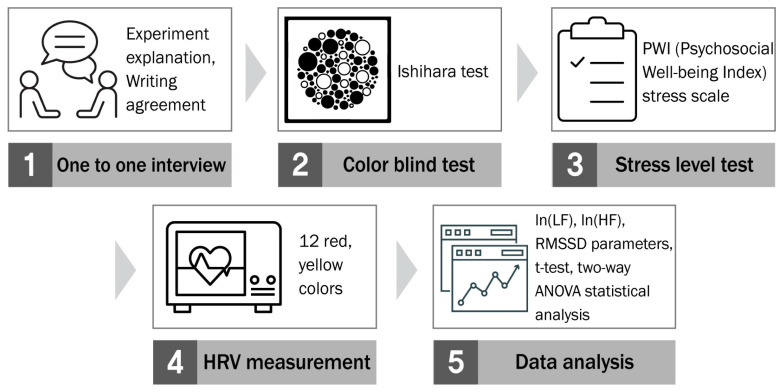
Experimental procedure.

The experiment was conducted in five steps (Figure 5). Steps 1, 2 and 3 corresponded to a pre-experimental stage. Step 1 was a one-on-one interview with the participant. Step 2 examined the participants who consented to participate in the study for color blindness or color weakness. Step 3 assessed the participant’s stress level by using the PWI stress scale. Step 4 measured HRV in a color environment. At step 5, we carried out a t-test statistical analysis on the ln(LF), ln(HF), and RMSSD parameters. Focusing on the In(LF) parameter showing significant results, the interactions between hue, brightness, saturation, and the stress group were verified through a two-way ANOVA analysis.

Participants signed the consent form to participate in the experiment after the researcher summarized the experiment and things to keep in mind. This experiment was conducted only for individuals who volunteered to participate. The Ishihara test was used to test the participants for color blindness or color weakness. None of the participants had color blindness or color weakness. 

The PWI was used to assess participants’ daily stress levels and assign participants to the stress groups. The participants who completed the stress level test underwent HRV measurement in a color environment; HRV was measured with the participants staring at 12 different colors in red and yellow color groups in the order of exposure. 

The measurement device used in this study for HRV was a uBio Macpa (Table 8), which fulfills the HRV measurement and analysis guidelines proposed by the Task Force of the European Society of Cardiology and the North American Society of Pacing and Electrophysiology. The ANS data standards proposed by the American HeartMath Institute were applied to this device system. It is a device with a measurement range of 40–200 beats per minute (BPM) and a measurement error of 2%. It is used to measure heart rate status [47], and its reliability has been proved by a number of papers applying this device and software [47,48,49]. The analysis software used in this study (Table 9) is a professional program capable of converting raw data into Excel Application; this software can extract data related to parameters such as mean pulse, stress index, complexity, Total Power, VLF, LF, HF, LF/HF, BPM, SDNN, and RMSSD.

**Figure 6:**
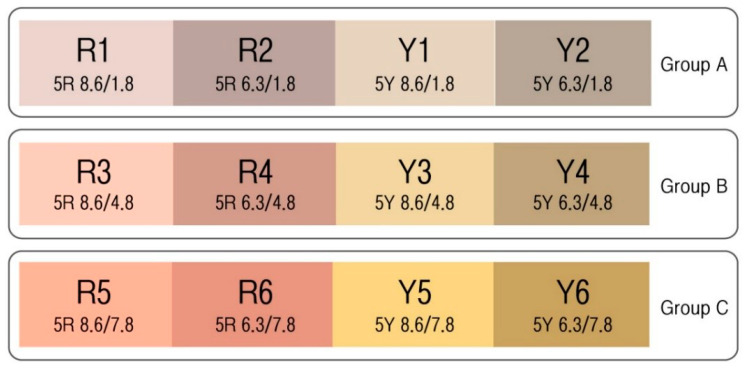
Order of exposure to experimental stimulus materials.

The order of exposure to the color stimulus material is presented in Figure 6. The order was determined by randomizing the materials for Groups A, B, and C [3]. Before the experiment, participants’ eyes were covered with an eye patch, and they were allowed to rest for 5 min [50]. They were then exposed to each of the experimental stimuli for 2 min and 30 s [51,52]. 

Subsequently, they were allowed to rest for 1 min while wearing the eye patch, and behaviors that can affect the ANS, such as deep breathing, sneezing, coughing, and yawning, were restricted during the experiment. 

The reason for limiting the measurement time to 2 min and 30 s is that if the measurement time was 5 min, the total experimental time would be 60 min after being exposed to 12 colors of stimulation. The long-term experiments can place the subjects under considerable psychological and stress stimulatory factors. Therefore, we decided that the ideal measurement time was 2 min and 30 s, which is the minimum amount of time required to measure HRV, according to a study by Berkoff et al. [51] and Munoz et al. [52]. However, we used RMSSD parameters as an analysis indicator to complement the limitations of short-term measurements [12,26].

After completing the HRV measurement experiment, we analyzed the data by examining the HF, LF, and RMSSD parameters of HRV. The graphs of LF and HF values were highly nonlinear at the lower frequency regions; thus, a natural log was used to correct it. The natural logs of the LF and HF spectral power of HRV, ln(LF), and ln(HF), respectively, were used to analyze the data [24,53,54]. The PWI scale distinguished the participants by their stress levels, and they were assigned to the potential stress group or the healthy group. The t-test statistical analytical method was used to examine how ln(LF), ln(HF), and RMSSD changed according to the environmental color changes.

## 3. Results and Discussion

### 3.1. Results

Table 10 shows the average value, standard deviation, and t-test results of ln(LF), ln(HF), and RMSSD in the two groups at each test color environment. 

In Table 10, there is no significant difference in ln(HF), RMSSD between the potential stress group and healthy group, and a statistically significant difference is observed in ln(LF) for red colors (e.g., R2 (t = −2.113; *p* < 0.05), R4 (t = −3.024; *p* < 0.01), and R5 (t = −3.366; *p* < 0.01)) and for yellow colors (e.g., Y1 (t = −2.960; *p* < 0.01), Y2 (t = −3.480; *p* < 0.01), Y3 (t = −2.316; *p* < 0.05), Y4 (t = −2.159; *p* < 0.05), and Y6 (t = −2.619; *p* < 0.05)). 

ln(LF) was significant for both groups; thus, we used ln(LF) for examining how the group results changed in response to a change in environmental color in detail. Two-way analysis of variance was performed to validate the major effects of stress and color on ln(LF) in each group and to validate the interaction between the color and stress groups. The analysis results of the ln(LF) values of the two groups according to the changes in hue, brightness, and saturation conditions are shown in Table 11, Table 12 and Table 13.

First, the ln(LF) values in the potential stress group and healthy groups based on the differences in colors when the saturation and brightness conditions were kept constant was analyzed using a two-way analysis of variance, and the results are shown in Table 11. The main effect of the stress group in terms of ln(LF) of the comparison of color was statistically significant overall, but not the main effect of the color. Moreover, no significant results could be discovered in the interaction effect between colors and the two groups. This indicates that the changes of colors, that is, the changes between the red color and yellow color, have little influence on ln(LF) of the potential group and the healthy group.

Second, the results of the two-way analysis of variance performed on the ln(LF) in the potential stress group and the healthy group, based on the differences in brightness when the color and saturation level were constant, are shown in Table 12. The main effect of brightness on ln(LF) is not statistically significant, and the main effect between the potential stress group and the healthy group is statistically significant. We did not observe any statistically significant interaction effect between the brightness and the two groups. This demonstrates that the effect the changes in brightness of color have on the ln(LF) values of the potential stress group and healthy group is small. 

Third, two-way analysis of variance of the ln(LF) values in the potential stress group and healthy group based on the differences in saturation when the color and brightness conditions were constant was performed, and the results are described in Table 13. The main effect of changes in the saturation on ln(LF) values was not statistically significant, but the main effect between the two groups was statistically significant. Additionally, there was no statistical significance found on the interactive effect between the saturation and two groups. Similar to brightness, this shows that saturation changes do not have a significant effect on the ln(LF) values in the potential stress group and healthy groups. 

### 3.2. Discussion

The literature has demonstrated that HF represents PNS activities and LF represents SNS activities. Although researchers have presented various hypotheses to explain the LF, the common consensus is that short-term LF is affected by SNS activities and PNS activities [55,56]. Although many researchers agree that the RMSSD parameter is generally valid in the extremely short-term measurement of less than 5 min, with the recent use of mobile device and applications to measure HRV values and estimate the stress level of the subjects, there have been increased discussions on parameters that have valid value in HRV measurements of below 5 min [57]. Salahuddin et al., 2007 [58], Castaldo et al., 2019 [59], Baek et al., 2015 [60], and Marek Malik et al., 1996 [26], verified that standardized LF and HF are common parameters that have valid measures in the extremely short-term measurement of HRV values less than 5 min. Based on our review of the literature, our study primarily used ln(LF), ln(HF), and RMSSD to analyze HRV changes due to color environment changes for each of the stress groups [29].

The value of ln(LF), known to reflect the activation of both the sympathetic and parasympathetic nerve in short-term measurements [55,56], was generally higher in the healthy group compared with that in the potential stress group. However, we could not accurately elucidate why the healthy group’s ln(LF) values were found to be higher than those in the other group. 

However, studies that observed LF increases in the healthy group under stress stimulation have reported that when recollecting the stressful events, the healthy control group showed increases in heart rate and LF, and LF did not increase significantly in the patients with posttraumatic stress [26,61]. This finding indicates that a significant degree of ANS overactivation could be sustained to slow the stress stimulus response in posttraumatic stress disorder. 

The result of analyzing RMSSD values which has validity in short-term measurements, [26,28] there was no intergroup statistically significant differences were noted, but the potential stress group showed a tendency of higher RMSSD levels. Considering that RMSSD, which is known to be positively correlated with stress conditions such as tension, depression, fatigue, and frustration. ln(LF) values were statistically higher in the healthy group compared with those in the potential stress group, which were divided based on the level of stress using the PWI assessment, and the average RMSSD parameters was higher in the potential stress group. These demonstrate the potential of the PWI scale to be utilized as a supporting tool to assess the stress level of the subjects. Two-way analysis of variance of ln(LF) values among the HRV parameters that showed statistical significance between the two groups based on each color was performed to confirm the main effects and interaction between the color condition (hue, brightness, saturation) and the two groups. The main effect of color conditions (hue, brightness, saturation) was not significant, and the main effect on each of the two stress groups was statistically significant overall at *p* < 0.05. Additionally, there was no statistically significant result found in the interaction effect between color conditions and the stress group.

From the results, it can be observed that setting the color stimulant at a point similar to the actual color environment does not have a significant effect on the HRV values of the two groups over a short-term period. Thus, the color condition applied in the actual environment is comparatively less stimulating visually and physiologically than the primary colors with high visual stimulus. Most of the previous research on the effect of color on subjects’ physiological responses [14,15,16,17,18,19,20,21] used primary colors with strong stimulant effects as test stimulants and showed statistically significant results. This study was different compared with such previous research in that it used color ranges that reflected the actual environment instead of primary colors as a test stimulant. There was no finding on interaction effect that showed that color, brightness, or saturation changes had effects on the two stress groups. 

Previous studies claimed that conditions of three factors of color, “hue”, “brightness”, and “saturation”, have physiological and psychological effects [62,63,64]; furthermore, it has specifically been proven that the low range of saturation induces stress and negative psychological effects [34,35,65]. Regarding brightness, Valdez and Mehrabia verified that the higher the brightness, the more effective it is in stimulating arousal [64], and Zielinski verified that arousal is affected by high brightness only with colors of high saturation [66]. In other words, high brightness of colors stimulates arousal, which revitalizes the body, thereby implying that high rather than low brightness colors reduce stress. Hence, we can observe that there is a high potential that a low saturation and low brightness range of color may induces stress. Another point to be discussed is the HRV values measurement time. In prior studies, the HRV values is generally measured for 5 min, and the guidelines also recommend 5 min as the measuring time [26]. However, in this study, the accumulative experiment time amounted to 60 min if the subjects were to be measured for 5 min for each color, and there was concern that the long experiment time may cause exhaustion and stress on the subjects. With the recent supply of mobile HRV devices, there has been some active research and discussions ongoing on the parameters that are at an extremely short-term measurement of below 5 min, and this study referred to some of the prior studies [58,59,60,61] to set the HRV value measurement time for each color at 2 min and 30 s. As a result, there was a statistically significant difference between the two groups in terms of ln(LF) values. However, further research is required to examine the fact that there was no statistical significance observed in terms of ln(HF) and RMSSD values. Our research indicates a potential for further validation on the test process exploring how the use of color stimulant with higher visually stimulating effect, and the longer measurement time of 5 min, obtained by minimizing the number of test color stimulants, changes the results of statistical analysis. 

However, the short HR measurement record (2 min and 30 s) and only analyzing the In(LF) parameter for stress evaluation are the major limitations of our study, thereby implying that it corresponds to basic research. We seek to complement such limitations in the future and conduct a further developed follow-up study.

Color is perceived by human vision and is a factor affecting emotions; an individual’s emotional status is an independent factor affecting the HRV [29]. Thus, emotional evaluation of color, in addition to the HRV measurement, will allow multifaceted interpretations of our study outcomes. 

Further research should conduct the HRV measurement and emotional assessment in response to color stimulation to identify physiological stress or psychological stress changes. Additionally, this study used red and yellow hues as experimental stimulus material. Further research is necessary to describe how HRV changes in response to color stimulus materials of various hues.

From an environmental design perspective, this study was conducted to identify specific color conditions that negatively affect individuals’ stress levels. Color is a space element perceived through human vision to affect the emotional aspect; thus, determining if a specific color has a positive or negative effect on stress is not a straightforward task. Our study applied the HRV measure to quantify stress due to environmental color and evaluated its efficacy. We posit that the findings of our study can be used as evidence for further research.

## 4. Conclusions

This study aimed to describe the effects of environmental color changes in hue, brightness, and saturation on HRV characteristics of groups differentiated by stress level. Our intention in this study was to provide an empirical base and evidence for designing a pleasant indoor environment by avoiding using the colors negatively affecting stress, at the time of environmental color planning, by identifying the environmental color conditions that affect stress status negatively.

Based on the findings, our conclusion are as follows. 

First, the ln(LF) of the healthy control group of a reduced stress level was higher than that of the potential stress group, indicating that the healthy control may have responded more sensitively to the stress stimulus than the potential stress group did. 

Second, the changes of environmental colors’ hue, brightness, and saturation were found to have no direct effect on the HRV parameters of the two stress groups. The short-term HRV measurement in environmental colors with weak visual stimulation compared with primary colors was found to have no influence on the stress of the two stress groups. To verify significant statistical results that hue, brightness, and saturation affect stress, environmental colors must be composed of colors with strong visual stimulation, and a long-term analysis of the HRV measurement record is considered to be required.

Third, statistically significant results were exhibited when the In(LF) parameter values of the two stress groups distinguished by the PWI index had identical hue, brightness, and saturation conditions. This finding suggests that only the stress factors of the two groups sorted by stress levels influence the HRV parameters, implying that the PWI index can be utilized as a reliable scale for measuring stress levels.

## Figures and Tables

**Table 1:** Parameters for time-domain and frequency-domain analyses of heart rate variability (HRV) signals.

Method	Parameter	Unit	Description
Time-domain Analysis	Mean HR	1/min	Mean heart rate
	Mean RR	Ms	Mean RR interval
	SDNN	Ms	Standard deviation of RR interval
	RMSSD	Ms	Square root of mean squared differences between successive RR intervals
Frequency-domain Analysis	pNN50	%	NNN50 divided by total number of RR intervals
	VLF	ms2	Absolute power of VLF band
	LF	ms2	Absolute power of LF band
	HF	ms2	Absolute power of HF band
	LF norm	n.u.	LF power in normalized units LF/(Total Power-VLF) × 100
	HF norm	n.u.	HF power in normalized units HF/(Total Power-VLF) × 100
	LF/HF	-	Ratio of LF band power to HF band power

**Table 2:** Studies of short-term HRV measurement with color as the experimental stimulus.

Authors	Experimental Variables	Number of Participants	Data Analysis Method	HRV Measurement	HRV Parameters that Changed Significantly	Study Limitations
Chäfer and Kratky [28]	Color lighting (red, green, blue; 700 lux)	*n* = 12 (average 29.9 years old)	T-test	NN, SDNN, RMSSD, VLF, LF, HF, LF/HF, DFA	VLF, HF, DFA	The study focused primarily on color light therapy; thus, the color light of high illuminance was used for the experiment.
Choi et al. [29]	Color lighting (blue, red, white)	*n* = 92 (56 men and 36 women; average 26.4 years old)	T-test, ANOVA	HF, LF, LF/HF, SDNN, RMSSD	HF, RMSSD	The experiment was conducted in color light of low illuminance not used in daily life. The color lighting was evaluated after the measurement of HRV.
Litscher et al. [30]	Color lighting (red, blue; 140 lux)	*n* = 7 (2 men and 5 women/average 34.1 years old)	T-test, ANOVA	HR, total HRV, LF/HF	HR, total HRV	In this pilot experiment, nasal temperature, heart rate, and HRV were measured.
Yuda et al. [31]	Color lighting (red, green, blue)	Experiment 1: *n* = 12 (10 men and 2 women; average 23 years old) Experiment 2: *n* = 4 (2 men and 2 women; average 23 years old)	T-test, ANOVA	LF, HF, LF/HF	HF, LF/HF	On the basis of the result from Experiment 1, in Experiment 2, HRV measurement was performed to verify that HF decreased in blue light.
Araujo et al. [32]	Color lighting (red, blue; 22–30 lux)	*n* = 5 (22–52 years old)	Shapiro test ANOVA	HR, RR, SDNN, RMSSD, LF, HF, LF/HF, SD1, SD2, ApEn, SampEN	HF, ApEn	HRV was measured in color lighting of low illuminance; it was found that photoplethysmography could measure HRV reliably.

**Table 3:** Specifications of the spectrophotometer (Minolta CM-2500d).

Model	Minolta CM-2500d	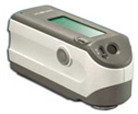
Illumination, viewing system	di:8, de:8 (diffuse illumination, 8-degree viewing), equipped with simultaneous measurement of SCI (specular component included)/SCE (specular component excluded) conforms to CIE No.15, ISO 7724/1, ASTM E1164, DIN 5033 Teil 7, and JIS Z8722 Condition C standard	
Wavelength range	360–740 nm	
Wavelength pitch	10 nm	
Reflectance range	0 to 175%, resolution: 0.01%	
Light source	Two pulsed xenon lamps	

**Table 4:** List of public health facilities for environmental data measurement.

Category	No.	Name of the Facility	Location	Total Area (m^2^)	Facility Size	Opening Year
General hospital	1	Gachon University Gil Hospital	Incheon	50,541	1691 beds	1987
	2	Seoul National University Bundang Hospital	Gyeonggi	127,727	1350 beds	2003
	3	Seoul National University Hospital	Seoul	252,009	1782 beds	1978
	4	Samsung Medical Center	Seoul	198,347	1979 beds	1994
	5	Seoul St. Mary’s Hospital	Seoul	190,000	1355 beds	2009
	6	Asan Medical Center	Seoul	280,991	2715 beds	2008
	7	Severance Hospital	Seoul	171,290	1263 beds	2005
	8	Ajou University Hospital	Gyeonggi	102,479	1088 beds	1994
Nursing hospital	9	Gangbuk Silver Welfare Center	Seoul	5381	100 beds	2013
	10	Yangcheon Municipal Elderly Care Center	Seoul	2533	80 beds	2011
	11	Bohyeon-haengwon Elderly Care Center	Gimhae	3424	80 beds	1994
	12	Municipal Elderly Care Center	Seoul	7940	258 beds	1995
	13	Sinmangae Elderly Care Center	Busan	3372	138 beds	2006
	14	Aegwang Elderly Care Center	Busan	3021	79 beds	1998
	15	Walker Hill Silver Town	Seoul	2146	70 beds	2011
	16	Hyoneung-won Elderly Care Center	Gimhae	2172	80 beds	2008
Public health clinic	17	Nowon-gu Public Health Center	Seoul	2424	4 floors	2011
	18	Seongbuk-gu Public Health Center	Seoul	906	4 floors	2011
	19	Bupyeong-gu Chungchun Public Health Center	Incheon	1089	3 floors	2011
	20	Buk-gu Gangbuk Public Health Center	Daegu	778	3 floors	2009
	21	East City Public Health Center	Gimhae	656	3 floors	2011
	22	Yeonje-gu Jaeban Public Health Center	Busan	805	4 floors	2010

**Table 5:** Data analysis of environmental colors in public health facilities.

Category	Range of Wall Color Data (Min–Max)		Average Color Data	
	Range of Brightness	Range of Saturation	Brightness	Saturation
Dominant color	5.3–9.5	0.3–4.2	8.6	1.8
Complementary color	5.2–10.2	0.1–6.6	7.4	2.5
Accent color	3.2–8.5	0.2–10.3	6.3	3.4

**Table 6:** Specifications of experimental environment artificial lighting (Osram FHF32SS EX-D).

**Model**	**Color Temperature**	**CRI**	**Light Output**	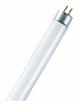
Osram FHF32SS EX-D	6500 K	60	2850 lm	

**Table 7:** Specifications of photometer (Luton LM-81LX).

**Model**	**Measurement**	**Measurement Range (Accuracy)**		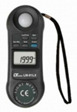
Lutron LM-81LX	Lux, foot-candle	Lux	Foot-Candle	
		0–20,000 Lux (±5% rdg ±8 dgt)	0–2000 Fc (±5% rdg ±8 dgt)	

**Table 8:** Specifications of the measurement device for HRV (uBio Macpa).

**Model**	**Power**	**Power Consumption**	**Dimension**	**Measurement Range**	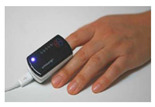
uBio Macpa	220 V, 60 Hz for computer system and 5V-DC through USB for measurement probe	0.25 W	58 mm (W) × 27 mm (H) × 32 mm (D)	40–200 BPM	

**Table 9:** Specifications of the software application for HRV.

Model	CPU	OS	Memory	Disk	Measurement Screen
uBio Macpa Software Application	Pentium IV 1 GHz above	Windows 2000 Windows XP, Windows Vista, Windows 7/8	1 GB above	100 GB above	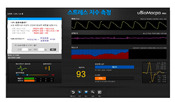

**Table 10:** Average ln(LF), ln(HF), and RMSSD values and t-test result subject to each environmental color for different stress groups.

R Colors					Y Colors				
Classification		Potential Stress	Healthy	*p*	Classification		Potential Stress	Healthy	*p*
R1 (5R 8.6/1.6)	ln(LF)	7.41(0.64)	7.83(0.87)	0.154	Y1 (5Y 8.6/1.8)	ln(LF)	7.31(0.82)	8.19(0.71)	0.007 **
	ln(HF)	6.67(0.65)	6.65(0.69)	0.929		ln(HF)	6.69(0.67)	6.67(0.53)	0.933
	RMSSD	34.60(17.44)	31.96(12.62)	0.663		RMSSD	35.56(13.02)	33.29(12.90)	0.655
R2 (5R 6.3/1.8)	ln(LF)	7.19(0.71)	7.73(0.58)	0.045 *	Y2 (5Y 6.3/1.8)	ln(LF)	7.37(0.52)	8.06(0.49)	0.002 **
	ln(HF)	6.53(0.70)	6.58(0.51)	0.838		ln(HF)	6.60(0.57)	6.59(0.50)	0.969
	RMSSD	35.31(14.84)	30.18(11.49)	0.335		RMSSD	34.86(12.31)	32.45(10.91)	0.600
R3 (5R 8.6/4.8)	ln(LF)	7.37(0.74)	7.78(0.64)	0.148	Y3 (5Y 8.6/4.8)	ln(LF)	7.30(0.62)	7.83(0.54)	0.029 *
	ln(HF)	6.61(0.67)	6.63(0.73)	0.966		ln(HF)	6.61(0.67)	6.63(0.51)	0.96
	RMSSD	37.73(22.67)	29.03(9.57)	0.226		RMSSD	34.82(13.55)	29.77(9.49)	0.285
R4 (5R 6.3/4.8)	ln(LF)	7.39(0.58)	8.04(0.52)	0.006 **	Y4 (5Y 6.3/4.8)	ln(LF)	7.47(0.61)	7.97(0.58)	0.041 *
	ln(HF)	6.53(0.65)	6.58(0.50)	0.834		ln(HF)	6.54(0.69)	6.52(0.48)	0.922
	RMSSD	36.03(16.75)	30.37(11.02)	0.323		RMSSD	36.89(18.65)	30.39(9.28)	0.282
R5 (5R 8.6/7.8)	ln(LF)	7.19(0.50)	7.94(0.67)	0.002 **	Y5 (5Y 8.6/7.8)	ln(LF)	7.52(0.41)	7.78(0.58)	0.195
	ln(HF)	6.63(0.61)	6.69(0.66)	0.793		ln(HF)	6.69(0.53)	6.62(0.65)	0.76
	RMSSD	37.30(13.53)	32.43(10.78)	0.320		RMSSD	38.13(14.44)	30.45(10.72)	0.138
R6 (5R 6.3/7.8)	ln(LF)	7.61(0.60)	7.86(0.72)	0.345	Y6 (5Y 6.3/7.8)	ln(LF)	7.41(0.59)	8.08(0.72)	0.015 *
	ln(HF)	6.62(0.59)	6.63(0.59)	0.983		ln(HF)	6.61(0.51)	6.71(0.60)	0.66
	RMSSD	34.18(10.29)	29.61(11.20)	0.280		RMSSD	36.89(12.94)	32.04(10.16)	0.298

Notes: ln(LF): log of low frequency heart rate variability; ln(HF): log of high frequency heart rate variability; RMSSD: root mean square of successive RR interval differences; *p* < 0.01 **, *p* < 0.05 *.

**Table 11:** The main effect and interaction by hue changes and stress groups of ln(LF).

Color	Variable	Sum of Squares	Degrees of Freedom	Mean Square	F	*p*
R1 × Y1	Color	0.222	1.000	0.222	0.384	0.538
	Stress Group	5.735	1.000	5.735	9.903	0.003 **
	Color × Stress Group	0.700	1.000	0.700	1.209	0.277
	Error	28.955	50.000	0.579		
R2 × Y2	Color	0.901	1.000	0.901	2.608	0.113
	Stress Group	4.988	1.000	4.988	14.436	0.000 ***
	Color × Stress Group	0.072	1.000	0.072	0.207	0.651
	Error	17.278	50.000	0.346		
R3 × Y3	Color	0.002	1.000	0.002	0.004	0.947
	Stress Group	2.862	1.000	2.862	6.929	0.011 *
	Color × Stress Group	0.051	1.000	0.051	0.123	0.728
	Error	20.654	50.000	0.413		
R4 × Y4	Color	0.000	1.000	0.000	0.000	0.996
	Stress Group	4.396	1.000	4.396	13.241	0.001 **
	Color × Stress Group	0.073	1.000	0.073	0.221	0.640
	Error	16.599	50.000	0.332		
R5 × Y5	Color	0.093	1.000	0.093	0.319	0.574
	Stress Group	3.400	1.000	3.400	11.731	0.001 **
	Color × Stress Group	0.833	1.000	0.833	2.875	0.096
	Error	14.493	50.000	0.290		
R6 × Y6	Color	0.001	1.000	0.001	0.002	0.963
	Stress Group	2.740	1.000	2.740	6.394	0.015 *
	Color × Stress Group	0.579	1.000	0.579	1.350	0.251
	Error	21.426	50.000	0.429		

*p* < 0.001 ***, *p* < 0.01 **, *p* < 0.05 *.

**Table 12:** The main effect and interaction by brightness changes and stress groups of ln(LF).

Measure	Variable	Sum of Squares	Degrees of Freedom	Mean Square	F	*p*
R1 × R2	Color	0.359	1.000	0.359	0.722	0.399
	Stress Group	3.104	1.000	3.104	6.239	0.016 *
	Color × Stress Group	0.042	1.000	0.042	0.084	0.774
	Error	24.876	50.000	0.498		
R3 × R4	Color	0.274	1.000	0.274	0.693	0.409
	Stress Group	3.675	1.000	3.675	9.304	0.004 **
	Color × Stress Group	0.203	1.000	0.203	0.513	0.477
	Error	19.750	50.000	0.395		
R5 × R6	Color	0.393	1.000	0.393	1.025	0.316
	Stress Group	3.333	1.000	3.333	8.693	0.005 **
	Color × Stress Group	0.867	1.000	0.867	2.261	0.139
	Error	19.173	50.000	0.383		
Y1 × Y2	Color	0.015	1.000	0.015	0.035	0.853
	Stress Group	8.216	1.000	8.216	19.236	0.000 ***
	Color × Stress Group	0.133	1.000	0.133	0.312	0.579
	Error	21.357	50.000	0.427		
Y3 × Y4	Color	0.317	1.000	0.317	0.905	0.346
	Stress Group	3.502	1.000	3.502	10.005	0.003 **
	Color × Stress Group	0.002	1.000	0.002	0.006	0.939
	Error	17.503	50.000	0.350		
Y5 × Y6	Color	0.125	1.000	0.125	0.372	0.545
	Stress Group	2.801	1.000	2.801	8.363	0.006 **
	Color × Stress Group	0.551	1.000	0.551	1.646	0.205
	Error	16.746	50.000	0.335		

*p* < 0.001 ***, *p* < 0.01 **, *p* < 0.05 *.

**Table 13:** The main effect and interaction by saturation changes and stress groups of ln(LF).

Measure	Variable	Sum of Squares	Degrees of Freedom	Mean Square	F	*p*
R1 × R3 × R5	Color	0.047	2.000	0.024	0.051	0.950
	Stress Group	5.571	1.000	5.571	12.099	0.001 **
	Color × Stress Group	0.518	2.000	0.259	0.563	0.572
	Error	34.534	75.000	0.460		
R2 × R4 × R6	Color	1.308	2.000	0.654	1.677	0.194
	Stress Group	4.555	1.000	4.555	11.673	0.001 **
	Color × Stress Group	0.580	2.000	0.290	0.743	0.479
	Error	29.265	75.000	0.390		
Y1 × Y3 × Y5	Color	0.466	2.000	0.233	0.591	0.556
	Stress Group	6.160	1.000	6.160	15.627	0.000 ***
	Color × Stress Group	1.332	2.000	0.666	1.689	0.192
	Error	29.568	75.000	0.394		
Y2 × Y4 × Y6	Color	0.014	2.000	0.007	0.020	0.980
	Stress Group	7.578	1.000	7.578	21.828	0.000 ***
	Color × Stress Group	0.135	2.000	0.068	0.195	0.823
	Error	26.038	75.000	0.347		

*p* < 0.001 ***, *p* < 0.01 **.

## Data Availability

No additional data available.
